# Effects of Physical Exercise on Prosocial Behavior of Junior High School Students

**DOI:** 10.3390/children8121199

**Published:** 2021-12-18

**Authors:** Yi Wan, Yue Zhao, Honglian Song

**Affiliations:** Department of Physical Education, Nanjing Forestry University, Nanjing 210037, China; wanyi@njfu.edu.cn (Y.W.); zhaoyue@njfu.edu.cn (Y.Z.)

**Keywords:** sports participation, prosocial behavior, OLS estimation, PSM estimation

## Abstract

Objective: This study aims to investigate whether physical activity can promote students’ prosocial behavior by analyzing the relationship between sports participation and the prosocial behavior of junior high school students. Methods: Based on the 2014–2015 China education panel survey (CEPS), the relationship between regular athletic sports and prosocial behavior was evaluated among eighth-grade students by ordinary least squares (OLS) estimation and propensity score matching (PSM) and the relationship between OLS and PSM was evaluated by Rosenbaum’s bounds test for a robustness test; the observations were analyzed for heterogeneity to identify those who benefit more from sports. Results: The OLS results showed that sporting behavior increased prosocial behavior scores by 4%, and the PSM results showed that regular physical exercise increased students’ prosocial behavior tendencies by over 0.2 standard deviations from the mean prosocial behavior score (standardized). According to the Rosenbaum’s bounds test, the estimates were robust and reliable, and the results of the heterogeneity analysis showed that with males and students whose fathers had more than 9 years of education, the students showed more significant improvements in prosocial behavior. Conclusion: Physical activity has a significant positive effect on students’ prosocial behavior, and gender and the father’s education are significantly related to prosocial behavior.

## 1. Introduction

There is currently a movement in China that aims to strengthen physical exercise programs for primary and secondary school students and to enhance their physical fitness. In 2020, the Central Committee of the Communist Party of China (CPC) announced Opinions on deepening the integration of sports and education to promote the healthy development of youth [1]. In the Opinions, the CPC further emphasized the need to strengthen school sports and to assist students in enjoying physical exercise, improving their physique, developing their personality, and exercising their will. Moreover, the central government’s emphasis may reflect the current severe lack of physical activity among youth and adolescents.

The significant contribution of physical exercise to the physical fitness and mental health of students has been well-documented. Available evidence also indicates that physical exercise is a promising strategy for mental health promotion and early intervention, which has been confirmed during the COVID-19 pandemic [2,3,4]. However, relatively little is known about the effects of physical exercise on the prosocial behaviors of students. The existing studies indicate that physical exercise helps us to acquire a series of personal and social skills that can improve psychosocial aptitudes and the ability to adapt to the social environment [5,6]. Furthermore, the Charity Aid Foundation reports that the altruistic behaviors of Chinese citizens, such as voluntary service, donation, and helping others, are at a low level worldwide in 2021 [7]. It is urgent to improve the prosocial behaviors of Chinese citizens. The adolescent stage is a critical period for the development of individual prosocial behaviors, and meanwhile, prosocial behaviors are also one of the important factors affecting the development of adolescents. Thus, it is of great significance to explore the effects of physical exercise on the prosocial behavioral tendencies of students.

Prosocial behavior is an important aspect of an individual’s fitness. Previous research has indicated that prosocial behavior is important in determining adolescents’ academic performance, interpersonal relationships, physical and mental health development, and socialization [8,9,10,11,12]. It has also been found that a person’s prosocial preference significantly improves their labor market performance. Further, Van Tongeren and Green [13] have studied the altruistically motivated prosociality and provided initial evidence that prosociality enhances meaning in life. Moreover, Layous et al. [14] have demonstrated that a person’s prosocial behavior plays an important role in social adaptation and social harmony.

For the individual, prosocial behavior helps to improve self-esteem [15], bring a sense of meaning [16], cope with negative emotions such as anxiety and loneliness [17], increase individual happiness [18] and life satisfaction [19], and obtain a higher level of mental health. From a social perspective, prosocial behavior is an important foundation for the construction and maintenance of a harmonious society and promotes the progress of social civilization [20]. It indicated that prosocial behavior is a symbol of public ethics, social responsibility, and public welfare, and it effectively promotes the harmonious development of society and is essential to the development of human society. Thus, the identification of strategies that improve individuals’ prosocial tendencies is an important issue.

According to current research, there are numerous avenues via which physical exercise may improve the prosocial behavior of individuals. Physical exercise increases the opportunity for and frequency of interpersonal interactions and affects individual mental health. First, physical exercise can increase empathy, promote collective participation, and increase interpersonal trust, thereby enhancing individual prosocial behavior [21]. Second, physical exercise improves mental health leading to a more optimal emotional state, which facilitates the implementation of prosocial behaviors. For example, previous studies have found that physical exercise can significantly reduce depression and increase happiness [22,23,24]. Moreover, physical exercise may increase interpersonal interactions, thereby promoting prosocial behavior tendencies [25]. Chaddock et al. found that physical exercise for at least 60 min a day can effectively improve the anti-interference ability of adolescents aged 9–12 [26]. Moreover, Hu et al. surveyed 526 people who regularly participate in physical exercise or rarely exercise. Dividing items into different groups showed that team projects and moderate-intensity sports can have a more positive impact [27]. Experts have also studied the physiological and effects of the psychological mechanism of exercise on the emotional state, indicating that exercise alleviates anxiety, stress, and depression [28]. Further, Malm C et al. suggested that even modest exercise is superior to being inactive or sedentary [29]. Therefore, sports should have a positive impact on many aspects, such as the degree of individual socialization and social adaptability. Notably, most of the existing research findings on physical exercise concern the positive impact of team exercise, whereas experimental research is more focused on interventional methods. Moreover, the mechanism influencing physical exercise and prosocial behavior should be analyzed.

Prosocial behavior is motivated by many individual and environmental factors. The individual level includes four aspects, namely, empathy [30], viewpoint selection [31], emotion [32], and personality [33]. At the level of environmental factors, both family and school markedly influence prosocial behavior [34], where environmental factors usually include social interactions with family members and other individuals.

Previous studies have investigated the relationship between physical activity and individuals’ prosocial behaviors. However, most studies have only considered the effects of prosocial preferences on physical activities. For instance, a previous investigation found that prosocial behavior plays an important role in physical activities. The authors verified the effectiveness of youth sports activities by analyzing participation behaviors and prosocial behaviors based on youth sports activities [35]. García-García et al. assessed a personal and social responsibility program in terms of students’ and their families’ perceptions of prosocial behaviors and physical activity levels [36]. Griese et al. found that in low-income communities, prosocial behavior plays an important role in the ability of individuals to engage in health-promoting behaviors such as sports activities [37].

The purpose of this study is to use the China Education Panel Survey (CEPS) to analyze the influence of regular participation in physical exercise on students’ prosocial behaviors and investigate the use of physical exercise as a means to develop prosocial behavior in junior high school students.

## 2. Materials and Methods

### 2.1. Data

The data used in this article originate from the 2014–2015 (CEPS), which was implemented by the China Survey and Data Center of Renmin University of China (RUC). This is the first continuous and large-scale follow-up survey project on young students, starting from the junior high school stage in our country. The survey adopted a probability sampling method proportional to size, whereby 4 schools in 28 counties (districts) were randomly selected according to grade (first grade and third grade of junior high school). The data covered 112 schools, 438 classes, and approximately 20,000 students nationwide. The subjects of the survey include students, parents, teachers, and school leaders. The data were mainly based on students, and various factors such as students’ basic individual characteristics, family characteristics, school characteristics, and students’ cognitive and non-cognitive abilities were investigated. We use the “cognitive ability test”, which includes the three cognitive tests of attention, memory, and reasoning ability, to test students’ cognitive ability.

The main advantage of the use of the CEPS in this study is that it has investigated both students’ physical exercise behaviors and prosocial behaviors. Regarding their physical exercise behavior, the CEPS asked students “You usually do physical exercises __ days a week, __ minutes a day”. Since 5 days is the upper 75% cut-off point for the frequency of participating in physical exercise, if a student performed physical exercise on more than 5 days a week, they were considered in the current study to regularly participate in physical exercise.

Regarding prosocial behavior, CEPS asked, “In the past year, did you improve the following points?” “Helping the elderly do things”, “Obeying orders, consciously queuing up”, and “Being sincere and friendly to others”. The answers included five options, namely, “never”, “occasionally”, “sometimes”, “often”, and “always”, which were assigned 1, 2, 3, 4, and 5 points, respectively. Following common psychological practice, the answers to the three questions and the obtained total prosocial behavior score were summed. Then, the internal reliability coefficient was calculated using Cronbach’s α coefficient, which was 0.77, indicating relatively good internal reliability. The scores were standardized in the course of the estimation.

Herein, we process the data as follows. Students whose daily exercise time was in the 99% quantile were excluded. These students exercised more than 6 h a day on average, and the longest exercise duration was even longer than 24 h, indicating that the data may contain errors. Then, urban, rural, and residential household registration samples were distinguished to determine whether students from different household types obtain different benefits from physical activity. Finally, after deleting missing values, 7666 observations were recorded, including 2760 regular participants and 4906 non-regular participants.

It is evident that there are huge differences between urban and rural families in our country. Such family differences may be reflected in the differences in students’ behaviors. China’s household registration system has been loosened in recent years, and some areas have carried out household registration reforms, unifying urban and rural household registrations into residential household registrations. Even so, the difference between urban and rural areas remains and will not disappear soon after the unified household registration. Moreover, household registrations differ from rural and urban household registrations.

The main control variables in this study included basic demographic characteristics of the students, namely, gender, age, ethnicity, body mass index (BMI), cognitive ability, number of siblings, father’s education, and mother’s education. Because the students were all eighth graders, it was not necessary to control the education stage. The statistical description of the main analyzed variables is shown in Table 1.

**Figure 1:**
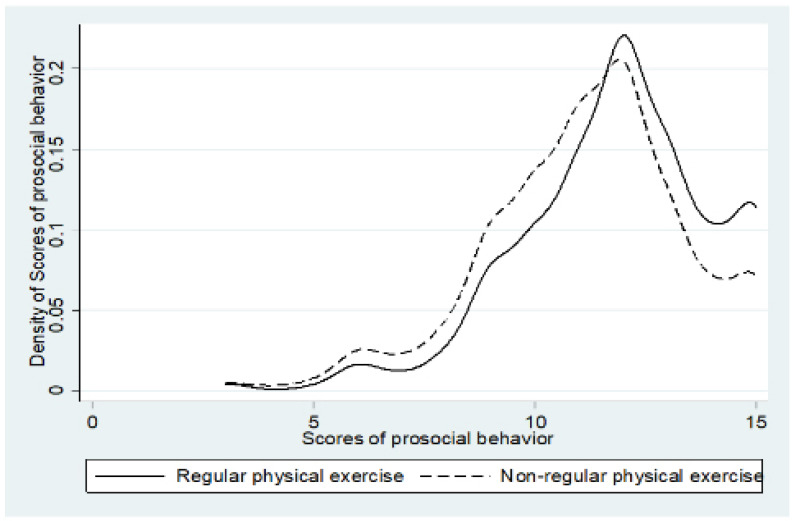
Distribution of prosocial behavior scores with regards to the regular participation in physical exercise.

The distribution of prosocial behavior scores with regard to the regular participation in physical exercise is presented in Figure 1. The prosocial behavior scores for students who regularly participate in physical exercise were obviously more concentrated in the high range, while the scores for those who do not often participate in physical exercise were more concentrated in the low range. This shows that regular participation in physical exercise can significantly improve students’ prosocial behavior.

### 2.2. Identification Strategy

We used a linear model to estimate the impact of physical exercise:

(1) prosocpref=α+βsport+γX+ε

(2) sport=I[πX+v>0].

Here, prosocpref represents the score of a student’s prosocial behavior, whereas sport indicates whether the student participates in sports. If the student participates in physical exercise, its value is 1; otherwise, it is 0. X represents other control variables. ε and v are the error terms. β is the influence coefficient for relevant sports. We expected that sports would have a significant positive impact on students’ prosocial behavior.

Notably, if the impact of physical exercise is estimated directly, there may be estimation bias caused by selection bias. Students who regularly participate in physical exercise may be individuals who enjoy social activities, master better social skills, and have higher prosocial behavior tendencies. Those students who do not or rarely participate in physical exercise are people who do not like social interactions; therefore, they have low prosocial tendencies, indicating bias in the estimation results. The traditional method to solve this problem is to determine the instrumental variables that affect students’ participation in physical exercise but do not affect their prosocial tendencies. A credible instrument variable must meet the following two criteria. First, it should directly influence students’ regular participation in physical exercise. Second, it should be strictly exogenous, not directly affecting the dependent variables, and having no direct causal relationship with the dependent variables.

The proportion of students of other ages in the school who regularly participate in physical exercise can be used as an instrumental variable to determine whether students often participate in physical exercise. There are three main factors influencing students’ regular participation in physical exercise. The first factor is students’ personal preferences. If a student enjoys physical exercise, he or she will participate in it often. The second factor is the sports facilities of the school. Participating in physical exercise requires certain prerequisites, such as the existence of a running track and a basketball court. The third is the degree of exercise participation. If individuals in a student’s surroundings do not like physical exercise, the student’s enthusiasm for regular participation in physical exercise may be affected. The proportion of students in other classes who regularly participate in physical exercise reflects whether the entire school has basic physical exercise facilities and whether there is a positive climate that encourages participation in physical exercise. However, the instrumental variables processed in this way cannot exclude the influence of community noise. Studies have shown that the neighborhood effect has a very important impact on children’s performance. Thus, it may not be possible to select strictly exogenous variables.

Considering these limitations, we used propensity score matching (PSM) to estimate the causal relationship between physical exercise and students’ prosocial behavior. Specifically, PSM aims to identify two groups of students who match each other. One group often participates in physical exercise, whereas the other does not. Then, their average prosocial behavior levels are compared.

Suppose that we use the 0–1 variable *sport* to distinguish whether students often participate in physical exercise: if the student often participates in physical exercise, the value of *sport* is 1; otherwise, its value is 0. For a student who regularly participates in physical exercise, we defined their potential prosocial behavior as *propref*_1_. The average processing effect of regular participation in physical exercise on students’ prosocial behavior performance is expressed as the difference between their actual prosocial behavior performance *E*(*propref*_0_|*sport* = 1) and their prosocial behavior performance *E*(*propref*_0_|*sport* = 1) under the assumption that they do not regularly participate in physical exercise, namely:

(3) $$ATT=E(propref_{1}|sport=1)-E(propref_{0}|sport=1)$$

Our sample is a cross-sectional one, and, therefore, we could only observe students in one state. In other words, we could observe the actual prosocial behavior of students who often participate in physical exercise, but we could not observe their infrequent participation in physical exercise. The PSM approach is to use the prosocial behavior of a student who matches the student but who does not regularly participate in physical exercise for the student’s potential prosocial behavior. An intuitive matching method is to match based on observable personal characteristics and then to analyze. However, when there are additional feature variables for matching, direct matching may encounter the problem of the “dimension curse”. Therefore, Rosenbaum and Rubin [38] proposed that the probability of an individual entering the processing group (in this study, indicating whether or not they often participate in physical exercise) can be estimated based on the individual’s characteristic information, and then the probability can be matched. Because matching changes from multiple dimensions to one dimension, the efficiency of matching is greatly increased, and the matching results are basically the same. The probability used here for matching is also called the propensity score, and this designates the origin of the name PSM.

To enable the use of PSM, two assumptions must be met in the current study. The first is the assumption of conditional independence. That is to say, control variable X may not only affect the decision of whether students often participate in physical exercise but also the performance of students’ prosocial behavior. Nonetheless, the decision of whether students often participate in physical exercise cannot affect these decision variables. Therefore, after controlling for decision variables, whether students often participate in physical exercise is random, and the difference in student behavior originates from the processing of whether students often participate in physical exercise. The second is the joint support hypothesis. The common support hypothesis requires that students with certain characteristics must have a positive probability of whether they do or do not often participate in physical exercise. This indicates that the probabilities of students participating in physical exercise under different conditions must overlap. This second hypothesis actually states that matching objects should be found among students who do not often participate in physical exercise. Otherwise, it would be impossible to analyze the effects of physical exercise. When the above two assumptions are satisfied, the difference in the performance of students’ prosocial behavior is caused by whether they often participate in physical exercise within the common support, namely:

(4) $$ATT=E\left[propref_{1}|sport=1,\;P\left(X\right)\right]-E\left[propref_{0}|sport=1,\;P\left(X\right)\right].$$

The last issue concerns the choice of matching methods, that is, how to match propensity scores. The matching methods that can be selected include nearest neighbor matching, radius matching, kernel matching, and local linear regression matching. The results obtained by using different matching methods should be consistent. Should different estimation results be obtained from different matching methods, this would indicate that the influence of physical exercise is uncertain. Therefore, looking at different matching methods from a certain angle can also be regarded as a robustness test.

We used Rosenbaum’s boundary estimation for sensitivity analysis. Rosenbaum’s boundary estimate calculates the average treatment effect of physical activity participation on a child’s performance when there are varying degrees of unobservable heterogeneity affecting physical activity participation. Before matching, there was some difference in the likelihood of physical exercise participation between the treatment group (individuals who regularly engage in physical activity) and the control group (individuals who did not regularly engage in physical activity). After matching for observable variables, if there is no unobservable heterogeneity affecting physical exercise participation, all of the individuals have equal propensity scores. If there is unobservable heterogeneity affecting physical exercise participation, then there is still a difference in the likelihood of physical exercise participation across individuals after matching observable variables. Rosenbaum’s bounds estimation tests whether a slight percentage increase in this difference would significantly change the estimates. The hypothesis test statistics were denoted as Γ. Γ = 1 means that the likelihood of physical exercise participation is the same. Γ > 1 means that different individuals differ in the likelihood of going out due to heterogeneity. By assigning different values to Γ, Rosenbaum’s bounds estimation gives the upper and lower significance levels of the impact of physical exercise participation at varying levels of variation on the likelihood of going out.

## 3. Results

### 3.1. Baseline Results: Ordinary Least Squares (OLS) Estimation 

We first used OLS to estimate the relationship between regular participation in physical exercise and the prosocial behavior of students. The estimated results are shown in Table 2 and indicate that there is a significant correlation between regular participation in physical exercise and the students’ prosocial behavior scores. Because we used standardized scores as dependent variables in the estimation, the results indicated that, on average, regular participation in physical exercise improved students’ prosocial behavior by 0.2 standard deviations. Given that the standard errors are about 2.2 in different samples, the results suggest that physical activity increased the prosocial behavior scores by 0.44 or 4%. After we controlled for various variables, the results were still significant. We also discovered that in different household registration samples, the effects of physical exercise were similar; however, the estimated coefficients in the household registration sample were slightly larger than those in the other two samples, although the difference was not significant.

Regarding the estimation results of other control variables, the variables with the greatest impact on students’ prosocial behavior include gender (female or not), cognitive ability, and father’s years of education. There was a significant positive correlation with students’ prosocial behavior scores for these three variables. Specifically, for a female student, the prosocial behavior score was, on average, approximately 0.2 standard deviations higher than that of a male student. For every 1% increase in cognitive ability (in logarithms), the student’s prosocial behavior score increased by approximately 0.3 standard deviations from the average. For each additional year of education a father received, his child’s prosocial behavior score increased by approximately 0.02 standard deviations. The effects of other variables were not robust and could therefore be ignored.

### 3.2. Balance Test

The reliability of the matching results also depends on whether the post-matching processing group and the control group are comparable, i.e., whether their basic characteristics are similar. The similarity of the basic characteristics between the treatment group and the control group was assessed with a balance test. The idea of the balance test is that the mean values of characteristic variables of the treatment group and the control group should not be significantly different after matching.

Table 3 reports the results of the balance test, with the treatment group and control group representing regular and infrequent participation in physical exercise, respectively. Further, Table 3 shows that before matching, there were significant differences between the treatment group and the control group with regards to other variables in addition to gender and the BMI index, indicating that a direct comparison between them may lead to larger estimation errors. After matching, there was no significant difference between the treatment group and the control group for all of the variables; this showed that matching eliminated observable heterogeneity, which subsequently resulted in comparable treatment and control groups.

### 3.3. PSM Results

Before the PSM estimation, we first had to estimate the tendency of individuals to regularly participate in physical exercise. Here, OLS was generally used; then, we could obtain the influencing factors that affected the students’ participation in physical exercise. The estimated results are presented in Table 4.

Table 4 shows that the main effective factors for regular participation in physical exercise are personal cognitive ability and the educational level of the father. However, the estimation results varied in different samples. In rural household registration, the main factors that affected whether a student often participates in physical exercise were his or her cognitive ability and the father’s education years. In the urban household registration, the main factor was the student’s cognitive ability. In the residential household registration, the influencing factors included the individual’s health level (BMI index), the mother’s years of education, and the father’s years of education.

**Figure 2:**
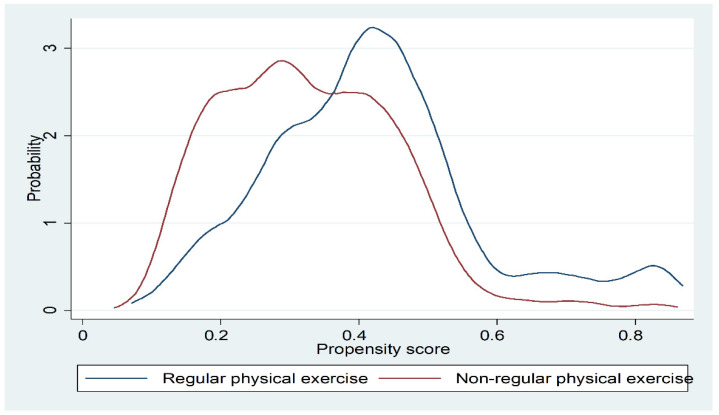
Propensity score distribution with regards to regular participation in physical exercise.

Based on estimating the influencing factors for regular participation in physical exercise, we obtained the propensity score for whether each student participated in physical exercise. Figure 2 shows the distribution of propensity scores for whether individuals often participate in physical exercise. Moreover, Table 2 demonstrates that the tendency scores for regular participation in physical exercise were overall more concentrated in the high range. However, there were obvious overlaps between the two, showing that the common support hypothesis for propensity score estimation is valid. Thus, PSM could be used to estimate the impact of regular participation in physical exercise.

Then, we used PSM to estimate the impact of regular participation in physical exercise. We employed four types of matching methods, namely, 1–1 matching, radius matching, kernel matching, and local least squares regression matching. The caliper value of 1–1 matching and radius matching was 0.01. The bandwidth of kernel matching and local least squares regression matching was also 0.01. Using different kinds of matching methods should yield similar findings if the results are robust. The estimated results are shown in Table 5, which indicates that regular participation in physical exercise significantly improves students’ prosocial behavior. On average, regular participation in physical exercise increased students’ prosocial behavior tendency by more than 0.2 standard deviations from the average prosocial behavior scores (standardized). This finding was very close to the OLS estimation result. Further, the estimation results using samples of rural households, urban households, and residential households were nearly the same.

### 3.4. Robustness Test

Finally, it is necessary to test the robustness or sensitivity of the estimated results. The Rosenbaum boundary test results are presented in Table 6. The Γ reflects the disturbance ratio. When Γ=1, this designates that there is no disturbance. When Γ=1.1, this indicates that the disturbance increases by 10%, and so forth. Table 6 shows that when Γ=1.9, the upper limit significance level is 6.02%. The upper limit significance level is below 1% when Γ<1.9, and the lower limits of the confidence intervals are greater than zero. This indicates that only a very large disturbance caused by unobservable heterogeneity would lead to significant differences in the estimated findings and render the results of physical exercise participation insignificant. This, in turn, shows that the estimation results in this study are robust.

### 3.5. Heterogeneity Analysis

To examine who would receive greater benefit from exercise, we divided the samples into two subsamples in terms of gender and the father’s education. Considering that the median of the father’s education was 9 years, we used 9 years of schooling as the threshold and divided the sample into two subsamples denoted as “low education” and “high education”. The results of the heterogeneity analysis are presented in Table 7; they indicate that male students benefited significantly more than female students. Similarly, students from families with fathers who had a high education experienced considerably more improvement in their prosocial behaviors.

## 4. Discussion

This study has focused on the relationship between physical activities and junior high school students’ prosocial behavior. Specifically, the study sought to explore whether physical exercise encourages students’ prosocial behavior, which may effectively promote the improvement of their physique and comprehensive qualities. This investigation has two main findings. First, OLS and PSM estimates suggest that physical exercise has significant and positive effects on students’ prosocial behavior. Second, males and students whose fathers’ schooling was longer than 9 years experienced greater improvement in their prosocial behavior.

First, our study confirms that physical exercise has significant and positive effects on students’ prosocial behavior. We used OLS and PSM to estimate the relationship between regular participation in physical exercise and the prosocial behavior of eighth-grade students. The OLS estimation results showed a significant correlation between regular physical exercise and students’ prosocial behavior scores. Specifically, regular physical exercise could increase students’ prosocial behavior by 0.2 standard deviations, and this significant correlation existed widely in samples with different household accounts. Previous studies have also referred to the positive and significant associations between physical exercise and prosocial behavior. Thus, higher participation in physical activities boosted the development of prosocial behaviors related to psychological well-being among school children [39]. Moreover, O’Donnell et al. explored the impact of sports activities on children’s prosocial behavior through comparative experiments and found that the group that participated in physical exercise improved their prosocial behavior, including their abilities with regards to emotional control, interpersonal relationships, and adaptation in school [40]. Chen et al. conducted a 6-month sports intervention on 100 rural left-behind children and found that the average score of the prosocial behavior dimension increased significantly after the intervention [41]. Notably, previous studies were largely based on intervention contrast experiments or discussed specific populations. However, our study, which is premised on the 2014–2015 National Educational Panel Survey, is based on objective data observations.

Second, our results indicate that gender (female or male), cognitive ability, and the father’s years of education are the variables with the greatest impact on students’ prosocial behavior. Following the above findings, we used PSM estimation and detected results that were very similar to the OLS estimation findings. Our previous estimates indicated matching analyses that were based on observable heterogeneity. However, there might be unobservable heterogeneity that affects the tendency of a student to participate in physical exercise, leading to unreliable results. We used the Rosenbaum boundary test to examine whether the estimation results can be maintained when a heterogeneity disturbance exists. The findings of the robustness test indicated that the estimation results observed in this study are robust. Further, we conducted a heterogeneity analysis to determine who benefits more from physical activity. A heterogeneity analysis by gender and the father’s education showed that males and students whose fathers’ schooling is longer than 9 years have more pronounced improvement in their prosocial behavior.

These findings are, in some regards, consistent with previous research results. In recent years, an increasing number of prosocial studies have consistently suggested that physiological factors, temperamental characteristics, socialization factors, social cognition, and other factors interact to jointly affect prosocial behavior. Gender is one of the factors that have previously been associated with prosocial behavior. Thus, an examination of gender prosocial behavior found that the effects of gender roles on behavior are mediated via hormonal processes, social expectations, and individual dispositions [42]. Another study detected higher prosocial scale values in girls [43]. Our study had similar findings since female students’ prosocial behavior scores were approximately 0.2 standard deviation points higher than that of male students. Males may benefit more from physical activity due to the fact that they engage in physical activity more often than females. Thus, both our study and previous studies highlight the significance of considering gender when analyzing the effects of physical activity on prosocial behavior.

In our study, students whose fathers’ schooling was longer than 9 years experienced greater improvement in their prosocial behavior. Interestingly, previous studies have found that parental education significantly affects children’s involvement in more physical activity [44,45,46]. Previous research has also focused on the impact of positive experiences of family intimacy on prosocial behavior. Thus, Knafo et al. observed that the family environment affects prosocial behavior [47]. Moreover, Eisenberg et al. found that positive parenting can promote the development of prosocial behavior of children and adolescents in a variety of ways [48]. However, our study is novel as we also focused on the impact of parents’ years of education on prosocial behavior. We firmly believe that our conclusion regarding the impact of parents’ education on prosocial behavior will enrich research on the individual differences of prosocial behavior and underlying mechanisms.

Notably, we consider that the form and intensity of physical exercise will provide valuable information. We tentatively present the influence of physical activity on prosocial behavior; however, the form and intensity of physical exercise were not discussed in detail. The intensity of physical exercise was considered in a previous study, which also found a consistent association between prosocial behavior and physical activity but insisted that there was no relationship between prosocial behavior and high-intensity activity [49]. Further, another study pointed out that basketball is more beneficial for prosocial behavior than running [50]. Moreover, a previous investigation tried to explore additional factors and found that short episodes of mindful practice (7–15 min) can increase prosocial behaviors [51].

In summary, we have identified the significant impact of physical exercise on prosocial behavior. Further, we have discovered the impact of the fathers’ years of education on prosocial behavior. Thus, by studying the impact of physical exercise on middle-school students’ prosocial behavior, we can provide suggestions for the education and guidance of the students’ prosocial behavior and develop a new educational intervention strategy to improve the students’ prosocial behavior. Moreover, creating conditions and an atmosphere for physical training may be instrumental to enhancing students’ prosocial behavior. Notably, studying the impact of physical exercise on students’ moral qualities will also assist in increasing our understanding of the role of physical exercise in students’ lives and contribute to the relevant literature.

### Study Limitations and Future Research Directions

Although this investigation has revealed important findings, it also has limitations. First, the sample used and its size restrict the generalizability of the results; this study is not generalizable to the entire Chinese population but is limited to students in the eighth grade. Second, this was a transversal study that only presents a causal relationship based on the heterogeneity of observables. We cannot provide a strong causal relationship excluding the potential for reverse causality. However, the observed strong and significant relationships suggest the validity of our findings. Third, this study investigates sports and the prosocial behavior of specific groups, explains the impact of sports activities on junior high school students’ prosocial behavior, and directly discusses the relationship between sport participation and prosocial behavior, but the measure of physical exercise does not differentiate between different kinds of sports (e.g., team vs. individual sports; at home vs. at school) and the different intensities of sports. Its reliability needs to be improved. Future research should address these issues. Moreover, factors that affect prosocial behavior through sports participation should be discussed; for instance, social desirability might play a role in assessing both physical exercise and—especially—prosocial behavior. In the future, we will consider prosocial behavior as an outcome variable and establish a research model with other variables to explore the relevant factors affecting prosocial behavior and promoting the development of prosocial behavior.

## 5. Conclusions

It is generally believed that physical exercise promotes students’ physical health, mental health, and learning performance. In addition, physical exercise may also improve the moral quality of students, thereby promoting interpersonal relationships and further improving students’ performance in all aspects. Our current study found the significant implications of physical activity on prosocial behavior. We believe this will enrich the theoretical research on children’s physical and mental health.

The findings of this study show that encouraging students to participate in physical exercise leads to effective improvement of the harmony and learning efficiency of the entire group. Therefore, schools and class groups must create an atmosphere and conditions that promote students’ active participation in all aspects of physical exercise. The government can also play a positive social role with regard to sports by improving public sports services and organizing public sports activities. Moreover, it would be appropriate to further promote the physical exercise of junior high school students. Since physical exercise has been proven to affect students’ behavior and moral qualities, it may provide a reference for the formulation of school extracurricular activities. We hope that through our research, we can provide helpful references for junior high school students.

## Figures and Tables

**Table 1:** Statistical description of the analyzed variables.

Variable	Full Sample	Rural Household	Urban Household	Resident Household
Prosocial behavior	11.444	11.246	11.794	11.486
	(2.239)	(2.232)	(2.130)	(2.340)
Physical exercise	0.360	0.339	0.368	0.403
	(0.480)	(0.473)	(0.482)	(0.490)
Gender	0.485	0.474	0.530	0.454
	(0.499)	(0.499)	(0.499)	(0.498)
Age	11.521	11.625	11.376	11.448
	(0.689)	(0.756)	(0.565)	(0.606)
Han nationality	0.914	0.906	0.917	0.932
	(0.279)	(0.291)	(0.275)	(0.250)
BMI index	19.191	18.962	19.382	19.533
	(3.318)	(3.189)	(3.399)	(3.493)
Cognitive ability	3.116	3.078	3.186	3.120
	(0.312)	(0.319)	(0.272)	(0.330)
Number of siblings	0.700	0.956	0.360	0.488
	(0.819)	(0.844)	(0.613)	(0.765)
Mother’s years of education	9.737	8.430	11.445	10.845
	(3.302)	(2.857)	(2.973)	(3.351)
Father’s years of education	10.432	9.237	12.005	11.425
	(2.863)	(2.280)	(2.705)	(3.032)
Observed value	7666	4037	2098	1531

Note: Standard errors are presented in parentheses.

**Table 2:** OLS estimation results.

Variable	Full Sample	Rural Household	Urban Household	Residential Household
Regular physical exercise	0.214 ***	0.209 ***	0.197 ***	0.236 ***
	(0.028)	(0.040)	(0.039)	(0.055)
Female	0.189 ***	0.218 ***	0.120 ***	0.201 ***
	(0.023)	(0.034)	(0.043)	(0.046)
Age	−0.010	−0.003	−0.047	−0.006
	(0.016)	(0.022)	(0.038)	(0.039)
Han nationality	0.158 ***	0.126	0.127	0.267 *
	(0.058)	(0.088)	(0.095)	(0.154)
BMI index	−0.005	−0.005	−0.005	−0.007
	(0.003)	(0.004)	(0.006)	(0.007)
Cognitive ability	0.325 ***	0.358 ***	0.291 ***	0.228 **
	(0.051)	(0.060)	(0.086)	(0.110)
Number of siblings	−0.005	−0.003	−0.050	0.002
	(0.015)	(0.017)	(0.044)	(0.044)
Mother’s years of education	0.008 *	0.008	0.007	0.009
	(0.004)	(0.006)	(0.008)	(0.010)
Father’s years of education	0.016 ***	0.017 **	0.009	0.026 **
	(0.005)	(0.007)	(0.009)	(0.010)
Urban household	0.035			
	(0.036)			
Residential household	−0.001			
	(0.037)			
Constant	−1.352 ***	−1.554 ***	−0.552	−1.285 *
	(0.309)	(0.397)	(0.628)	(0.667)
Observed value	7666	4037	2098	1531
R-squared	0.084	0.090	0.073	0.097

Note: The standard error of the cluster at the school level is in parentheses. We also controlled for county-level virtual fixed effects. The *, **, and *** represent the significance at the level of 10%, 5%, and 1%, respectively. The BMI means Body Mass Index.

**Table 3:** Balance test.

Factors	Matching Status	Treatment Group	Control Group	Deviation Ratio (%)	T Statistics	Cohen’d
Gender	Before matching	0.494	0.480	2.7	1.13	0.027
	After matching	0.494	0.475	3.7	1.37	0.037
Age	Before matching	11.487	11.542	−8.1	−3.35 ***	−0.080
	After matching	11.487	11.492	−0.7	−0.29	−0.007
Han nationality	Before matching	0.930	0.905	9	3.71 ***	0.088
	After matching	0.930	0.935	−1.7	−0.7	−0.017
BMI index	Before matching	19.264	19.151	3.4	1.43	0.034
	After matching	19.264	19.262	0.1	0.02	0.001
Cognitive ability	Before matching	3.168	3.087	26.5	10.94 ***	0.260
	After matching	3.168	3.166	0.4	0.16	0.004
Number of siblings	Before matching	0.635	0.736	−12.5	−5.21 ***	−0.124
	After matching	0.635	0.655	−2.5	−1	−0.025
Mother’s years of education	Before matching	10.222	9.464	23.1	9.7 ***	0.229
	After matching	10.222	10.193	0.9	0.34	0.009
Father’s years of education	Before matching	10.876	10.182	24.4	10.26 ***	0.243
	After matching	10.876	10.828	1.7	0.62	0.017

Note: *** represent significance 1%.

**Table 4:** Estimation of the tendency to participate in physical exercise.

Factors	Full Sample	Rural Household	Urban Household	Residential Household
female	0.0081	0.0154	−0.0082	0.0060
	(0.0142)	(0.0207)	(0.0239)	(0.0245)
Age	0.0055	0.0040	−0.0008	0.0220
	(0.0105)	(0.0124)	(0.0168)	(0.0224)
Han nationality	0.0071	0.0248	−0.0262	0.0023
	(0.0283)	(0.0372)	(0.0432)	(0.0616)
BMI	−0.0005	0.0013	0.0024	−0.0074 ***
	(0.0016)	(0.0025)	(0.0032)	(0.0028)
Cognitive ability	0.1125 ***	0.1340 ***	0.1240 ***	0.0343
	(0.0265)	(0.0322)	(0.0451)	(0.0416)
Number of siblings	−0.0025	0.0014	−0.0215	0.0115
	(0.0084)	(0.0104)	(0.0182)	(0.0187)
Mother’s years of education	0.0025	−0.0013	0.0003	0.0110 **
	(0.0024)	(0.0032)	(0.0052)	(0.0049)
Father’s years of education	0.0083 ***	0.0098 ***	0.0003	0.0115 **
	(0.0025)	(0.0034)	(0.0055)	(0.0057)
Urban household	−0.0117			
	(0.0189)			
Residential household	−0.0046			
	(0.0201)			
Constant	−0.1601	−0.2561	−0.0327	−0.0724
	(0.1549)	(0.1895)	(0.2572)	(0.3295)
Observations	7666	4037	2098	1531
R-squared	0.0938	0.0834	0.1169	0.1459

Note: The standard error from the cluster to the school level is in brackets. We also controlled for county fixed effects. In order to save space, the estimation results are omitted here **, and *** represent significance at the level of 5%, and 1%, respectively. In Table 4, “BMI” refers to body mass index.

**Table 5:** PSM estimation results.

Matching Method	Full Sample	Rural Household	Urban Household	Residential Household
1 to 1 match	0.2046 ***	0.2537 ***	0.2473 ***	0.2604 ***
	(0.0449)	(0.0476)	(0.0819)	(0.0904)
Radius match	0.2274 ***	0.2283 ***	0.2121 ***	0.2397 ***
	(0.0253)	(0.0328)	(0.0509)	(0. 0760)
Kernel match	0.2254 ***	0.2306 ***	0.2110 ***	0.2425 ***
	(0.0244)	(0.0335)	(0.0435)	(0.0618)
Local least squares regression matching	0.2318 ***	0.2238 ***	0.2334 ***	0.2422 ***
	(0.0264)	(0.0349)	(0.0525)	(0.0575)

Note: The standard error of bootstrap 500 times is in brackets. The matching variable is the same as the OLS estimate. The radius of one-to-one matching, the radius of radius matching, the bandwidth of kernel matching, and the bandwidth of local least squares regression matching are all set to 0.01. *** represent significance at the level of 1%.

**Table 6:** Rosenbaum’s boundary test results.

Γ	Upper Limit of Significance Level	Lower Limit of Significance Level	Lower Limit of Confidence Interval	Upper Limit of Confidence Interval
1.0	0.0000	0.0000	0.2785	0.2785
1.1	0.0000	0.0000	0.2534	0.3017
1.2	0.0000	0.0000	0.2267	0.3236
1.3	0.0000	0.0000	0.1817	0.3717
1.4	0.0000	0.0000	0.1173	0.4287
1.5	0.0000	0.0000	0.0901	0.4579
1.6	0.0000	0.0000	0.0739	0.4761
1.7	0.0000	0.0000	0.0573	0.4921
1.8	0.0034	0.0000	0.0413	0.5070
1.9	0.0602	0.0000	0.0259	0.5193
2.0	0.3241	0.0000	0.0091	0.5302

**Table 7:** Results of the heterogeneity analysis.

Matching Method	Female	Male	Low Education	High Education
1 to 1 match	0.1845 ***	0.2576 ***	0.1839 *	0.2640 ***
	(0.0529)	(0.0382)	(0.1041)	(0.0385)
Radius match	0.1711 ***	0.2283 ***	0.1952 ***	0.2456 ***
	(0.0380)	(0.0328)	(0.0509)	(0. 0760)
Kernel match	0.1783 ***	0.2536 ***	0.1941 ***	0.2486 ***
	(0.0339)	(0.0399)	(0.0380)	(0.0372)
Local least squares regression matching	0.1700 ***	0.2576 ***	0.2036 ***	0.2485 ***
	(0.0386)	(0.0382)	(0.0425)	(0.0475)
Observations	3724	3956	4269	3411

Note:The *, and *** represent significance at the level of 10%, and 1%, respectively.

## Data Availability

The data set contained personal information of the study participants. Our institutional review board will not have the provision to disclose any kind of information. Thus, our policy is not to make available the data set in the manuscript, the supplemental files, or a public repository. However, data related to this manuscript are available upon request, and researchers who meet the criteria for access to confidential data may contact Mrs. Yi Wan (wanyi@njfu.edu.cn).
